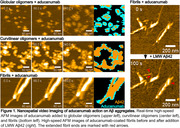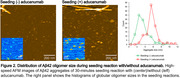# Nano‐space Video‐imaging reveals Action of ADUCANUMAB to Amyloid β aggregates: The Comparison with the Action of LECANEMAB

**DOI:** 10.1002/alz70861_108042

**Published:** 2025-12-23

**Authors:** Kenjiro Ono, Moeko Shinohara, Takahiro Nakayama

**Affiliations:** ^1^ Kanazawa University Graduate School of Medical Sciences, Kanazawa, Ishikawa Japan; ^2^ World Premier International Research Center Initiative (WPI)‐Nano Life Science Institute, Kanazawa University, Kanazawa, Ishikawa Japan

## Abstract

**Background:**

Antibody drugs targeting Aβ aggregation have been developed. Aducanumab has been approved by the FDA, and lecanemab and donanemab are on the market. We have demonstrated that real‐time observation of the binding of lecanemab to a single Aβ aggregate and single‐molecule structural dynamics analysis of antibody binding not only visualize the inhibition of the Aβ aggregation process by antibody drugs, but also help to understand the mechanism of action of antibody drugs (DOI: 10.1021/acs.nanolett.3c00187). In this study, we visualized the binding of aducanumab, which inhibits secondary nucleation (DOI: 10.1038/s41594‐020‐0505‐6), to Aβ42 aggregates at the single molecule level to compare the results of our research on “Lecanemab” with those on “Aducanumab”.

**Method:**

We prepared globular oligomers, curvilinear oligomers, and mature fibrils by in vitro aggregation of synthetic Aβ42. We placed these on mica and confirmed their shapes and sizes using high‐speed atomic force microscopy (HS‐AFM). Aducanumab was then added to the sample chamber in which the mica was immersed, and the binding of aducanumab to each aggregate on the mica was observed in real time with nanometer spatial resolution by HS‐AFM. In addition, low molecular weight (LMW) Aβ42 was applied to the aducanumab‐bound mature fibril and fibril elongation and seeding reaction were observed by HS‐AFM.

**Result:**

Aducanumab bound to and surrounded globular oligomers, curvilinear oligomers, and mature fibrils (left in Figure 1). When LMW Aβ42 was applied to aducanumab‐bound mature fibrils, larger oligomers formed in the presence of aducanumab (Figure 2) while some of the fibrils were observed to elongate (right in Figure 1).

**Conclusion:**

Aducanumab bound to spherical and curvilinear oligomers (protofibrils) and to fibrils. However, the amount of aducanumab bound to protofibrils was significantly lower than the amount bound to lecanemab. In addition, consistent with previous study results (DOI: 10.1038/s41594‐020‐0505‐6), aducanumab binding to fibrils did not significantly inhibit fibril elongation. However, we found that aducanumab promoted the formation of larger spherical oligomers in the seeding reaction, suggesting that the formation of larger oligomers may contribute to the inhibition of secondary nucleation.